# Hemodynamics and Metabolic Parameters in Normothermic Kidney Preservation Are Linked With Donor Factors, Perfusate Cells, and Cytokines

**DOI:** 10.3389/fmed.2021.801098

**Published:** 2022-01-10

**Authors:** Annemarie Weissenbacher, John P. Stone, Maria Letizia Lo Faro, James P. Hunter, Rutger J. Ploeg, Constantin C. Coussios, James E. Fildes, Peter J. Friend

**Affiliations:** ^1^Oxford Transplant Centre, Nuffield Department of Surgical Sciences, University of Oxford, Oxford, United Kingdom; ^2^Department of Visceral, Transplant and Thoracic Surgery, Medical University of Innsbruck, Innsbruck, Austria; ^3^The ex-vivo Lab, Division of Cell Matrix Biology and Regenerative Medicine, School of Biological Sciences, Faculty of Biology, Medicine and Health, The University of Manchester, Manchester Academic Health Science Centre, Manchester, United Kingdom; ^4^The ex-vivo Research Centre Community Interest Company (CIC), Macclesfield, United Kingdom; ^5^Institute of Biomedical Engineering, University of Oxford, Oxford, United Kingdom

**Keywords:** kidney transplantation, organ preservation, normothermic, urine recirculation, *ex-situ* perfusion

## Abstract

Kidney transplantation is the best renal-replacement option for most patients with end-stage renal disease. Normothermic machine preservation (NMP) of the kidney has been studied extensively during the last two decades and implemented in clinical trials. Biomarker research led to success in identifying molecules with diagnostic, predictive and therapeutic properties in chronic kidney disease. However, perfusate biomarkers and potential predictive mechanisms in NMP have not been identified yet. Twelve discarded human kidneys (*n* = 7 DBD, *n* = 5 DCD) underwent NMP for up to 24 h. Eight were perfused applying urine recirculation (URC), four with replacement of urine (UR) using Ringer's lactate. The aim of our study was to investigate biomarkers (NGAL, KIM-1, and L-FABP), cells and cytokines in the perfusate in context with donor characteristics, perfusate hemodynamics and metabolic parameters. Cold ischemia time did not correlate with any of the markers. Perfusates of DBD kidneys had a significantly lower number of leukocytes after 6 h of NMP compared to DCD. Arterial flow, pH, NGAL and L-FABP correlated with donor creatinine and eGFR. Arterial flow was higher in kidneys with lower perfusate lactate. Perfusate TNF-α was higher in kidneys with lower arterial flow. The cytokines IL-1β and GM-CSF decreased during 6 h of NMP. Kidneys with more urine output had lower perfusate KIM-1 levels. Median and 6-h values of lactate, arterial flow, pH, NGAL, KIM-1, and L-FABP correlated with each other indicating a 6-h period being applicable for kidney viability assessment. The study results demonstrate a comparable cytokine and cell profile in perfusates with URC and UR. In conclusion, clinically available perfusate and hemodynamic parameters correlate well with donor characteristics and measured biomarkers in a discarded human NMP model.

## Introduction

Improving the quality and duration of donor kidney preservation prior to transplant may increase utility and potentially improve outcomes. Machine perfusion is at the forefront of this field (1–4), but accurate monitoring and evaluation of the kidney is essential to optimize outcomes. Currently, no biomarkers exist that can predict the usability or quality of a kidney during perfusion. Broad hemodynamic parameters such as arterial flow during hypothermic machine perfusion (HMP) with DGF and higher levels of NGAL and/or L-FABP over time have been inversely associated with estimated glomerular filtration rate (eGFR) together with an increasing intrarenal resistance (IRR) (5). Critical criteria consisting of arterial flow, macroscopic appearance of the kidney and volume of excreted urine have been incorporated into a decision-making score during normothermic machine perfusion (NMP) (6), but clearly, a prognostic marker of post-transplant function would represent a stepwise improvement to perfusion.

The kidney is equipped with a sophisticated immune compartment, hosting a plethora of non-hematopoetic cell types and a variety of both transient and resident leukocytes (7, 8). This leukocyte population remains in a steady state until an immunological challenge occurs. In the transplant setting this consists of death in the donor, surgical intervention in the donor, preservation, and finally reperfusion in the recipient. A potent inflammatory cascade ensues involving severe cytokine activation and cellular extravasation (9–11). Given this inflammatory response is initiated in the donor and continues throughout preservation, evaluating leukocyte diapedesis and cytokine secretion may identify novel biomarkers during kidney perfusion.

On these grounds, the aim of this study was to investigate if perfusion inflammatory profiles correlate with (i) donor factors, (ii) perfusion hemodynamics, (iii) the type of volume management, applying either urine recirculation (URC) or urine replacement (UR), and (iv) biomarkers of renal injury (NGAL, KIM-1, and L-FABP) in a blood-based perfusate of long-term NMP (12, 13) of discarded human kidneys.

## Materials and Methods

### Donor Factors

Human kidney grafts, deemed not transplantable by all kidney transplant centers in the United Kingdom were included in this study. All organs were retrieved for the purpose of transplantation but discarded during post-procurement assessment. Donor and retrieval characteristics, kidney function parameter, and ischemia times were collected.

### NMP Perfusion

After being sent to Oxford, perfusions were performed at the Institute of Biomedical Engineering, University of Oxford. The technique of NMP using discarded human kidneys was reported previously (12, 13). Briefly, hemodynamic (arterial blood flow, mean arterial pressure and IRR) and biochemical perfusion parameters were recorded. Perfusate samples were collected and processed by centrifugation at 4,000 rpm for 15 min at 4°C. The supernatant was aliquoted, snap-frozen and stored at −80°C. The study was evaluated and approved by the National Ethics Review Committee of the United Kingdom (REC reference 12/EE/0273 IRAS project ID 106793).

### Volume Management

Continuous urine recirculation or urine replacement with Ringer's lactate was applied as reported previously (13, 14). Ringer's lactate was infused to replace the excreted urine as a 1:1 volume replenishment in 20 mL intervals (14). The pH was adjusted through titration with sodium bicarbonate 8.4% (5–15 mL) to the physiological level of 7.3 before kidney NMP was started. No additional sodium bicarbonate was given at any point during perfusion after kidney connection.

### Biomarkers of Renal Injury

Neutrophil gelatinase-associated lipocalin (NGAL), kidney injury molecule-1 (KIM-1), and liver fatty acid-binding protein (L-FABP) levels in the perfusate samples were measured. Neutrophil gelatinase-associated lipocalin (NGAL), kidney injury molecule-1 (KIM-1), and liver-type fatty acid-binding protein (L-FABP) levels in the perfusate samples were measured by a quantitative sandwich enzyme immunoassay technique using NGAL and KIM-1 Quantikine ELISA kits (R&D systems, USA) and Human FABP1/L-FABP ELISA Kit (CMIC Co., Ltd., supplied by R&D systems) according to manufacturers' instructions (12).

### Inflammatory Profiling

#### Luminex® Analysis

A commercially available human 13-plex magnetic bead panel (Merck Millipore, Billerica, Massachusetts, USA) was used, following the manufacturer's protocol. The plate was read using a Bio-Plex 200 system (Bio Rad, Hertfordshire, United Kingdom). Thirteen cytokines and chemokines were assessed: Interferon (IFN)-γ, Interleukin (IL)-1α, IL-1β, IL-1RA, IL-2, IL-4, IL-6, IL-8, IL-10, IL-12p40, IL-12p70, granulocyte macrophage colony-stimulating factor (GM-CSF), and tumor necrosis factor (TNF)-α (12).

#### Flow Cytometry

Samples of perfusate (4 ml) were collected into EDTA vacutainers, 0.4 ml dimethyl sulfoxide (DMSO) was added and well-mixed; 2 ml of this solution was then transferred into a cryogenic storage vial, moved to a CoolCell® Cell Freezing Container and stored in a −80°C freezer. Immunophenotyping of the human perfusate samples was performed on a BD LSR II flow cytometer (Becton Dickinson, Oxford, United Kingdom). Leukocytes were identified and gated as CD45+ and their viability assessed using an eFluor™ 506 viability dye (eBioscience, California, USA). Following this, a panel of antibodies was utilized to characterize T helper cells (CD3ε+CD4α+), cytotoxic T cells (CD3ε+CD8α+), double-positive T cells (CD3ε+CD4α+CD8α+), double-negative T cells (CD3ε+CD4α-CD8α-), γδ T cells (γδ+), B cells (CD3ε-CD21+), classical monocytes (CD14+CD163-), non-classical monocytes (CD14+CD163+), immature neutrophils (6D10+2B2-), mature neutrophils (6D10+2B2+), mature eosinophils/basophils (6D10-2B2+), and natural killer cells (CD335+). Cells were treated with red blood cell lysing solution (BD Biosciences, United Kingdom), washed, and resuspended in 0.3 ml of staining buffer. A 20 ml quantity of e123count beads (eBioscience, California, USA) was added and samples were analyzed for 3 min. All gating strategies and analysis were performed using FlowJo version 10.0.6 (12).

### Data Analysis

The statistical testing was done with Graph Pad Prism 7 and IBM® SPSS® Statistics Version 25. A *p*-value of <0.05 was considered as statistically significant. Biomarker, Luminex® and flow results, donor and perfusion factors were analyzed using parametric and non-parametric tests, including Spearman rank correlation. The Bonferroni method was applied to correct for multiple testing in the correlation analyses.

## Results

Twelve discarded human kidneys, seven from donors after brain death (DBD) and five from donors after circulatory death (DCD), were perfused for a median (min-max) of 12.8 (6.1–24.1) h. Volume management was facilitated by replacement of the urine (UR) with Ringer's lactate in four (4/12, 33.3%) NMP kidneys, and urine recirculation (URC) was applied in eight (8/12, 66.7%) kidney perfusions.

Table 1A illustrates the demographics for perfused DBD and DCD kidneys including donor risk indices. The median (min-max) UK kidney donor risk index [UKKDRI, (16)] was 1.9 (1.1–2.87); only three kidneys had a UKKDRI < 1.35 (Table 1). Median (min-max) CIT was 20.5 (12.7–46.9) h, median (min-max) WIT was 12 (9–15) min for DCD kidneys. DBD kidneys experienced a shorter median (IQR) CIT with 17.5 (5.9) h compared to 22 (24.1) h in DCD kidneys, *p* = 0.05. Median (min-max) donor urine output prior to retrieval was 60 (10–350) ml/h. Median (min-max) donor serum creatinine and eGFR at the time point of retrieval were 64.5 (32–208) μmol/l and 81 (29–247) ml/min/1.73 m^2^.

**Table 1A:** Organ donor characteristics, procurement parameters, and reasons for discard of kidneys.

	**Age in years**	**Sex**	**BMI in kg/m^**2**^**	**Donor type**	**Serum creatinine ^**@**^retrieval in μmol/L**	**eGFR ^**@**^retrieval in ml/min/1.73 m^**2**^**	**WIT in minutes**	**CIT in hours + minutes**	**Hypertension**	**UKKDRI/KDRI**	**Reason for discard**
Kidney 1	59	Male	35.1	DCD	32	247	15	21 + 16	Yes	1.10/1.21	Arteriosclerosis
Kidney 2	60	Male	35.1	DCD	114	71	14	42 + 17	Yes	1.50/1.60	Poor perfusion
Kidney 3	44	Male	41.4	DCD	63	120	12	46 + 59	Yes	1.22/1.13	Biopsy findings
Kidney 4	66	Female	31.2	DBD	208	41	n.a.	15 + 9	No	1.96/1/49	Patchy perfusion
Kidney 5	70	Female	24.3	DBD	44	119	n.a.	17 + 30	Yes	2.02/1.83	Stenosis of renal artery
Kidney 6	74	Female	24.8	DCD	57	90	11	22	Yes	2.07/2.17	Lesion on partner kidney (monomorphic cell infiltration)
Kidney 7	71	Female	28.1	DBD	86	56	n.a.	46 + 47	Yes	1.85/1.98	Anatomy, long CIT
Kidney 8	78	Female	25.4	DBD	79	61	n.a.	18 + 22	Yes	2.87/2.38	Vascular damage
Kidney 9	71	Female	29.1	DBD	66	77	n.a.	21 + 4	No	2.02/1.67	Organ size
Kidney 10	47	Female	39.1	DBD	152	29	n.a.	12 + 41	No	1.21/1.09	Vascular damage, patchy perfusion
Kidney 11	62	Female	23.5	DCD	62	85	9	19 + 52	No	1.67/1.50	Suspicion of cancer
Kidney 12	76	Female	24.5	DBD	36	152	n.a.	15 + 26	Yes	1.97/1.28	Long CIT

*BMI, body mass index; DCD, donation after circulatory death; DBD, donation after brain death; eGFR, estimated glomerular filtration rate MDRD (modification of diet in renal disease); WIT, warm ischemia time; CIT, cold ischemia time*.

*UKKDRI (15): Watson et al. (16); KDRI, OPTN KDRI/KDPI calculator*.

The median (min-max, IQR) duration of NMP was 12.8 (6.1–24.1, 17.2) h. The median (min-max) hourly urine output during NMP was 54.4 (1.7–471.9) ml/h and the median arterial flow was 370.8 (100–787) ml/min. The median (min-max) perfusate lactate during NMP was 12.8 (4.4–20) mmol/l and the median (min-max) perfusate pH throughout the preservation period was 7.36 (7.16–7.62). A significantly longer NMP period could be achieved in kidneys with URC (*n* = 3 URC kidneys 12 h, *n* = 1 URC kidney 18 h, *n* = 4 URC kidneys 24 h); median (IQR) NMP time of 21 (11.4) hours with URC vs. 7.2 (3) hours with UR (*n* = 2 UR kidneys 6 h, *n* = 1 UR kidney 8 h, *n* = 1 UR kidney 9 h), *p* = 0.01.

To compare similar adequate time points between URC and UR kidneys, the time interval for perfusate analyses was the start of NMP until hour 6 of NMP as all of the perfused kidneys reached at least 6 h of NMP. Table 1B summarizes the hemodynamic and metabolic function parameters for the individual kidneys. A total of 135 perfusate samples were analyzed; 45 per assessment of NGAL/KIM-1/L-FABP, cytokines, and cells.

**Table 1B:** Hemodynamic and metabolic function parameters.

	**Kidney 1**	**Kidney 2**	**Kidney 3**	**Kidney 4**	**Kidney 5**	**Kidney 6**
Arterial pressure in mmHg (mean, SD)^*^	84.6 ± 1.1	82.5 ± 7.8	91.3 ± 6.4	90.3 ± 1.3	90.4 ± 2.1	88.9 ± 1.7
Arterial flow in ml/min (mean, SD)^*^	650 ± 191.6	294.5 ± 74	325 ± 127.7	271 ± 54.6	383.9 ± 88.3	474.1 ± 149.7
IRR in ml/min/mmHg (mean, SD)^*^	0.15 ± 0.08	0.3 ± 0.09	0.39 ± 0.36	0.36 ± 0.18	0.25 ± 0.08	0.2 ± 0.09
pH (mean, SD)^*^	7.39 ± 0.12	7.21 ± 0.1	7.24 ± 0.17	7.33 ± 0.06	7.4 ± 0.04	7.66 ± 0.2
Arterial pO_2_ in kPa (mean, SD)^*^	15 ± 1.6	14.7 ± 0.7	12.6 ± 2	12 ± 1.5	13.8 ± 0.6	15 ± 3
Venous pO_2_ in kPa (mean, SD)^*^	7.7 ± 1.9	7.6 ± 0.8	6.4 ± 1.1	7 ± 2.4	8 ± 0.9	7.4 ± 0.9
Arterial pCO_2_ in kPa (mean, SD)^*^	4.3 ± 0.9	5.1 ± 0.4	5.9 ± 0.7	4.6 ± 0.7	5.1 ± 0.9	4.6 ± 0.6
Lactate level in mmol/l (mean, SD)^*^	13.4 ± 1.62	13.27 ± 1.32	12.19 ± 3	18.52 ± 2.16	5.65 ± 3.8	9.62 ± 4
Total glucose given in gram^**^	2.4	3	1.2	0.75	3.2	0.55
Total urine output in ml	828	286	10	105	1,285	445
Total urine output in ml/hour	63.7	15.9	1.7	8.8	53.5	74.2
Urine recirculation yes/no	Yes	Yes	Yes	Yes	Yes	No
Time on the device (hours + min)	13 + 1	18 + 3	6 + 20	12 + 35	24 + 5	6 + 10
	**Kidney 7**	**Kidney 8**	**Kidney 9**	**Kidney 10**	**Kidney 11**	**Kidney 12**
Arterial pressure in mmHg (mean, SD)^*^	92.4 ± 2.9	92.2 ± 2.8	89.8 ± 0.5	91.64 ± 2.3	89.2 ± 2.2	90.3 ± 2.1
Arterial flow in ml/min (mean, SD)^*^	148.2 ± 46.3	123.5 ± 79.16	339.6 ± 83.4	240.7 ± 120.9	468.8 ± 82.8	664.9 ± 228.1
IRR in ml/min/mmHg (mean, SD)^*^	0.76 ± 0.5	1.2 ± 1.02	0.3 ± 0.12	0.5 ± 0.3	0.19 ± 0.05	0.17 ± 0.13
pH (mean, SD)^*^	7.18 ± 0.08	7.2 ± 0.1	7.6 ± 0.2	7.33 ± 0.1	7.39 ± 0.04	7.43 ± 0.02
Arterial pO_2_ in kPa (mean, SD)^*^	13.6 ± 1.1	14.6 ± 2	14 ± 3.2	13.5 ± 2.9	15.4 ± 3.6	12.45 ± 0.8
Venous pO_2_ in kPa (mean, SD)^*^	8.3 ± 1.5	7.8 ± 1.8	6.4 ± 1.3	6.7 ± 1.3	7.1 ± 1.6	7.6 ± 1.3
Arterial pCO_2_ in kPa (mean, SD)^*^	5.4 ± 0.9	4.8 ± 1.1	5 ± 0.7	4.5 ± 0.9	4.7 ± 0.6	4.6 ± 0.4
Lactate level in mmol/l (mean, SD)^*^	16.18 ± 2.14	14.28 ± 4.5	16.34 ± 2.9	18.84 ± 2.2	9.19 ± 2	7.04 ± 1.46
Total glucose given in gram^**^	1	1.5	0.5	5	3	4.55
Total urine output in ml	50	920	675	11,325	1,325	1,223
Total urine output in ml/hour	8.3	102.2	84.4	471.9	55.2	51
Urine recirculation yes/no	No	No	No	Yes	Yes	Yes
Time on the device (hours + min)	6 + 5	9 + 25	8 + 10	24+5	24	24

* *Time-averaged longitudinal mean value compiled from hourly measurements over the course of the perfusion*.

** *Circulating perfusate volume of 500 ml*.

*Parts of donor and perfusion characteristics of kidneys 6, 8 and 9 without urine recirculation have been published previously (13)*.

**Figure 1:**
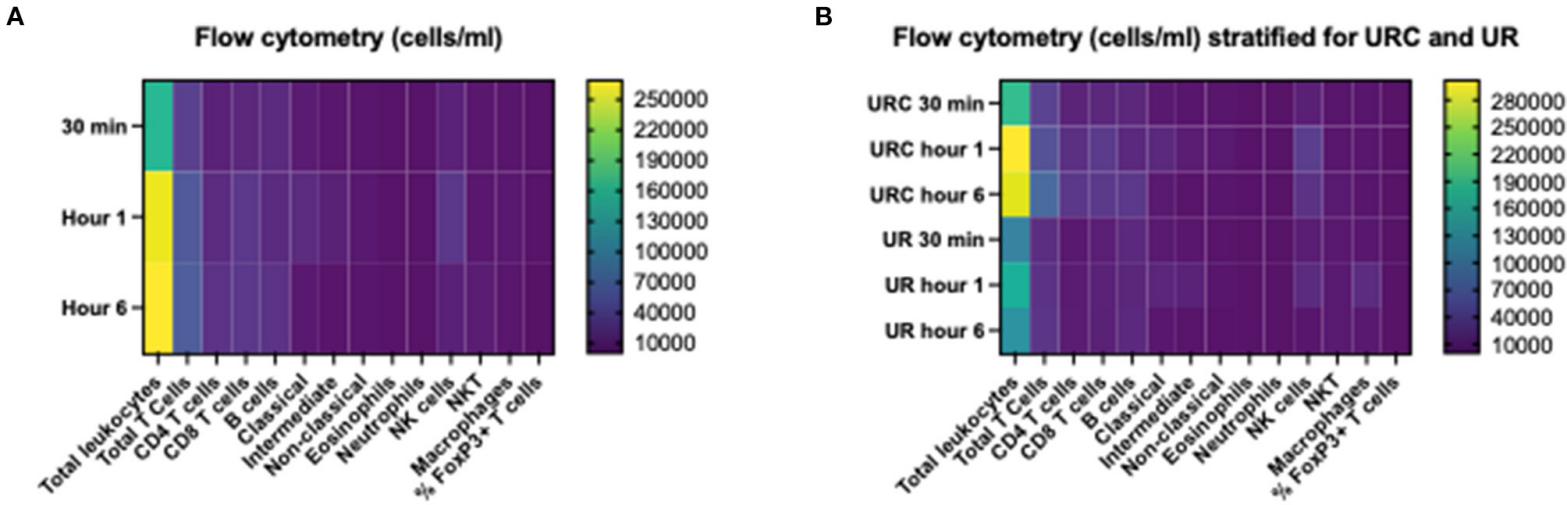
**(A)** Heatmap visualizing results of flow cytometry analyses (cells/ml perfusate, 500 ml overall volume) of *n* = 12 NMP kidneys; time points investigated: 30 min, 1 and 6 h after NMP start. Median of cell count per measured entity per time point is displayed. **(B)** Heatmap visualizing results of flow cytometry analyses (cells/ml perfusate, 500 ml overall volume) stratified for urine recirculation (URC) and urine replacement (UR) kidneys; time points investigated: 30 min, 1 and 6 h after NMP start. Median of cell count per measured entity per time point is displayed.

Table 2 shows the first and the 6-h measurements, as well as the Δvalues for NGAL, KIM-1, and L-FABP in absolute numbers. Table 3A displays the flow cytometry analyses results for all (*n* = 12) perfused NMP kidneys for time points 30 min, hours 1 and 6 after NMP-start. There were significantly more T-cells, CD4+ and NKT-cells in the perfusate after 6 h of NMP compared to 30 min after NMP-start. The content of monocytes, the intermediate type, was also significantly higher at hour 6 compared to the early measurements after initiation of NMP (Table 3A). Perfusate volume in NMP-kidneys was either managed with URC (*n* = 8) or UR (*n* = 4); we compared the potential efflux of cells between these two groups, shown in Table 3B. In regards to cell type and number of cells in the perfusate, there were no significant differences detectable between URC and UR kidneys throughout 6 h of NMP. Supplementary Table 1A displays cells in the perfusate of URC kidneys up to 24 h. To visualize changes of the cell count in the perfusate over time, a heatmap comprising all 12 NMP kidneys is pictured in Figure 1A; the changes of the number of cells in perfusates of kidneys undergoing different volume management (URC or UR) are depicted in Figure 1B.

**Table 2:** Observed perfusate biomarker concentrations.

**NGAL in ng/mL with urine recirculation**	First time point^*^	Last time point^**^	**Delta**	Median (IQR)^****^
Kidney 1	28.3	37.2	8.9	37.2 (9.6)
Kidney 2	67.1	102.1	35	97.9 (32.1)
Kidney 3	19.1	43.1	24	31 (24)
Kidney 4	60.2	54.3	−5.9	59.8 (6)
Kidney 5	8.9	7.7	−1.2	12 (24.7)
Kidney 10	59.1	122	62.9	182.2 (152.2)
Kidney 11	10.4	32.9	22.5	36.8 (34.1)
Kidney 12	10.7	1.6	−9.1	16.1 (47.7)
**NGAL in ng/mL without urine recirculation**				
Kidney 6	17.3	7.4	−9.9	7.4 (10.1)
Kidney 7	100.9	117.2	16.3	103.5 (16.3)
Kidney 8	88.5	4.1	−84.4	4.2 (84.4)
Kidney 9	16.2	7.5	−8.7	6.9 (12.1)
**KIM-1 in pg/mL with urine recirculation**				
Kidney 1	348.5	518.1	169.7	518.1 (389.3)
Kidney 2	1,132.7	1,206.7	74.1	1,213 (77)
Kidney 3	397.6	932.1	552.5	655.8 (552.5)
Kidney 4	731.1	1,144.3	413.2	1,144 (438.9)
Kidney 5	170.5	429.8	259.4	724.2 (618.8)
Kidney 10	211.8	267.1	55.3	305.7 (168)
Kidney 11	73.9	436.5	362.6	518.2 (489.4)
Kidney 12	55.6	545.6	490	828.3 (892.4)
**KIM-1 in pg/mL without urine recirculation**				
Kidney 6	75.3	151.5	76.3	151.5 (99.5)
Kidney 7	244.5	635.9	391.4	521.8 (391.4)
Kidney 8	258.3	404.4	146.1	404.4 (211)
Kidney 9	401.6	518.9	150.7	489.1 (128)
**L-FABP in ng/mL with urine recirculation**				
Kidney 1	15	< detection limit^***^	−15	0^***^ (15)
Kidney 2	47.9	2.5	−45.4	4.8 (37.1)
Kidney 3	31.1	0.7	−30.4	15.9 (30.4)
Kidney 4	862.8	877.3	14.5	862.8 (21.7)
Kidney 5	< detection limit^***^	13.6	13.6	26.9 (39.7)
Kidney 10	70.2	41.8	−28.4	56 (20.1)
Kidney 11	88.4	138	49.6	138 (36.6)
Kidney 12	154.8	< detection limit^***^	−154.8	10.4 (111.6)
**L-FABP in ng/mL without urine recirculation**				
Kidney 6	289.2	369.5	80.3	357.2 (79.7)
Kidney 7	852.5	859.4	6.9	855.2 (6.9)
Kidney 8	1.8	27.3	25.5	22.4 (25.5)
Kidney 9	39.6	< detection limit^***^	−39.6	4.2 (39.6)

* *1 h after perfusion start*.

** *6 h after perfusion start*.

*** *Minimum detectable dose for assay <6.25 ng/ml (L-FABP)*.

**** *Time-averaged longitudinal value compiled from all measurements over the course of the perfusion; perfusate volume = 500 ml*.

*Some NGAL, KIM-1 and L-FABP measurements of kidneys 6, 8, and 9 without urine recirculation have been published previously (13)*.

**Table 3A:** Flow cytometry results^*^ of *n* = 12 NMP kidneys.

	30 min^**^	1 h^**^	6 h^**^	*p*-value^***^	*p*-value^****^
Total leukocytes	166,023, 158,326	259,628, 190,892	268,659, 355,959	0.10	0.15
Total T Cells	33,434, 30,009	53,971, 35,611	58,032, 74,672	0.14	0.03
CD4 T cells	11,716, 20,791	18,821, 18,157	24,095, 49,548	0.28	0.03
CD8 T cells	15,124, 15,919	27,610, 26,454	27,612, 25,313	0.13	0.10
NK T cells	4,332, 5,681	4,997, 4,677	7,932, 8,762	0.25	0.03
B cells	17,692, 27,406	17,736, 27,009	23,732, 40,729	0.82	0.31
Monocytes					
*Classical*	7,480, 6,085	18,017, 19,586	5,547, 10,488	0.04	0.96
*Intermediate*	4,099, 4,603	11,412, 11,400	1,363, 1,696	0.01	0.03
*Non-classical*	2,452, 2,423	5,341, 6,669	2,320, 4,276	0.01	0.97
Eosinophils	393, 786	439, 742	711, 1,473	0.68	0.23
Neutrophils	157, 249	359, 217	242, 1,263	0.84	0.99
NK cells	11,780, 40,888	26,342, 49,070	10,883, 41,349	0.46	0.88
Macrophages	3,529, 6,142	5,929, 18,386	3,883, 5,279	0.12	0.81

* *Number of cells in cells/ml; overall perfusate volume = 500 ml.*

** *Time after start of NMP, values in median and IQR (interquartile range)*.

*** *Comparison 30 min with 1 h values*.

**** *Comparison 30 min with 6 h values*.

**Table 3B:** Flow cytometry results^*^ stratified for urine recirculation and urine replacement.

	**Kidneys with urine recirculation (*****n*** **=** **8)**			**Kidneys without urine recirculation (*****n*** **=** **4)**			*p*-value^***^30 min	*p*-value^***^ 1 h	*p*-value^***^ 6 h
	30 min^**^	1 h^**^	6 h^**^	30 min^**^	1 h^**^	6 h^**^			
Total leukocytes	194,687, 169,462	301,435, 173,632	285,198, 314,757	112,250, 125,008	173,140, 224,651	131,250, 371,186	0.37	0.21	0.68
Total T Cells	39,778, 42,889	54,203, 18,377	79,875, 79,263	21,809, 28,131	26,523, 47,966	28,027, 69,184	0.28	0.15	0.28
CD4 T cells	14,579, 24,059	24,088, 14,444	30,468, 54,969	6,123, 9,731	8,402, 11,934	9,112, 40,426	0.28	0.07	0.57
CD8 T cells	16,913, 13,757	33,008, 19,555	31,977, 21,867	11,074, 18,395	13,174, 32,824	12,905, 23,162	0.15	0.15	0.11
NK T cells	2,943, 5,681	4,595, 3,800	8,050, 11,446	6,254 ± 3,241	7,870, 5,795	7,932, 5,671	0.68	0.37	0.93
B cells	17,692, 29,619	18,707, 26,760	31,089, 43,484	18,933, 64,538	16,183, 60,774	19,205, 271,378	0.68	0.99	0.93
Monocytes									
*Classical*	6,310, 10,731	18,017, 19,859	7,188, 11,711	7,480, 2,978	15,596, 19,757	5,547, 5,020	0.99	0.93	0.99
*Intermediate*	3,462, 5,310	9,255, 11,880	1,363, 2,799	4,180, 3,842	13,508, 13,273	1,292, 1,749	0.81	0.46	0.93
*Non-classical*	2,743, 3,285	7,667, 6,711	3,790, 3,645	1,810, 3,074	2,960, 2,963	1,114, 1,364	0.15	0.05	0.05
Eosinophils	545, 12,346	613.4, 5,528	1,122, 24,421	332, 764	296, 853	711, 653	0.57	0.28	0.81
Neutrophils	157, 202	379.1, 156.2	210.2, 351.4	105, 392	296, 438	1,039, 2,564	0.88	0.49	0.20
NK cells	11,780, 56,253	34,918, 63,636	26,187, 48,707	10,316, 12,633	19,607, 22,801	3,549, 5,731	0.57	0.21	0.07
Macrophages	3,073, 9,088	4,433, 11,636	3,709, 4,719	6,469, 6,217	21,035, 42,213	5,988, 15,180	0.68	0.21	0.46

* *Number of cells in cells/ml; overall perfusate volume = 500 ml*.

** *Time after start of NMP, values in median and IQR (interquartile range)*.

*** *P-value result of comparison with and without urine recirculation*.

**Figure 2:**
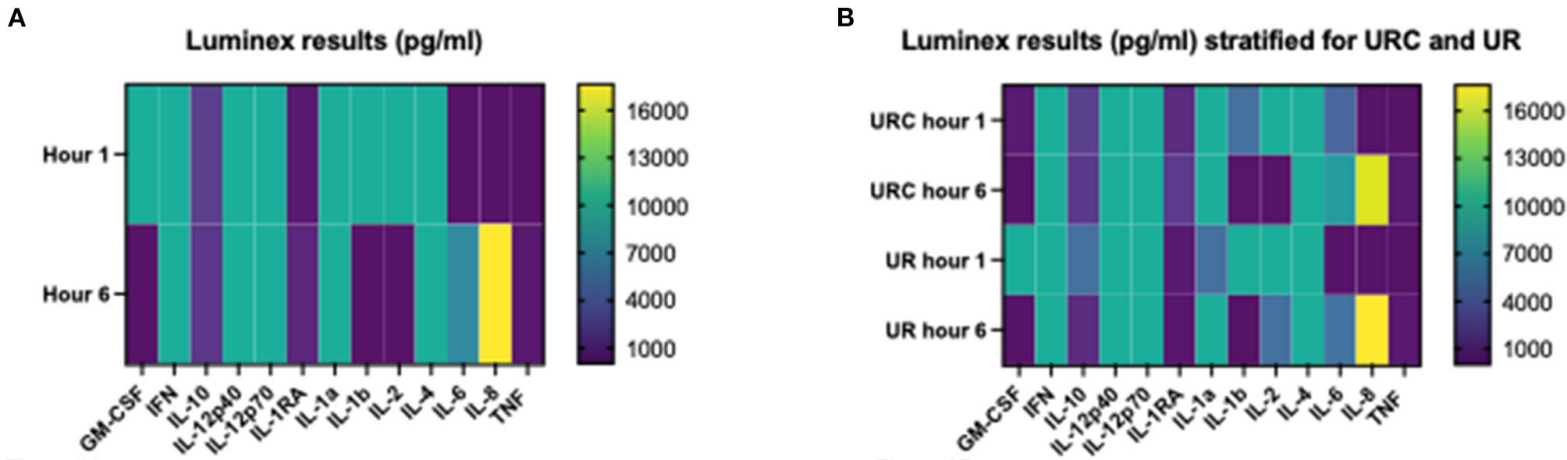
**(A)** Heatmap visualizing Luminex results (pg/ml) of *n* = 12 NMP kidneys; time points investigated: 1 and 6 h after NMP start. Median cytokine concentration per measured entity per time point is displayed. **(B)** Heatmap visualizing Luminex results (pg/ml) stratified for urine recirculation (URC) and urine replacement (UR) kidneys; time points investigated: 1 and 6 h after NMP start. Median cytokine concentration per measured entity per time point is displayed.

Table 4A shows the cytokine concentration measured in the perfusate after 1 and 6 h of NMP in the overall investigated cohort of 12 kidneys. Interleukin 8 was the only cytokine which increased significantly over time; *p* = 0.003, 95% CI of difference −21,204 to −2,633. Table 4B gives an overview of the potential effect of the type of perfusate volume management, URC or UR, on the concentration of cytokines in the perfusate. In both settings, URC and UR, IL-1ß decreased over time. There were no significant differences of ΔIL-1ß in perfusates of URC or UR kidneys between NMP-start and hour 6, *p* = 0.09. Supplementary Table 1B shows cytokines in the perfusate of URC kidneys up to 24 h. For visualization purposes, heatmaps for the cytokine concentration changes over time, were configured and are shown in Figures 2A,B. Figure 2A displays the development of cytokine changes over time in the overall cohort of 12 NMP kidneys, Figure 2B illustrates the stratification for URC and UR kidneys.

**Table 4A:** Luminex results^*^ of *n* = 12 NMP kidneys.

	1 h^**^	6 h^**^	***p*-value**
GM-CSF	10,000, 9,979	27, 208	0.77
IFN-γ	10,000, 0	10,000, 7,476	>0.9
IL-10	2,159, 9952	1,743, 3,646	>0.9
IL-12p40	10,000, 0	10,000, 7,481	>0.9
IL-12p70	10,000, 0	10,000, 0	>0.9
IL-1RA	453, 1,845	980, 1,997	>0.9
IL-1α	10,000, 7,472	10,000, 7,387	>0.9
IL-1β	10,000, 9,985	14, 164	0.94
IL-2	10,000, 7,497	20, 9,999	0.94
IL-4	10,000, 0	10,000, 0	>0.9
IL-6	45, 9,007	6,943, 4,882	0.89
IL-8	24, 872	17,625, 196,167	0.003
TNF-α	10, 214	398, 295	>0.9

* *Number of cells in pg/ml; overall perfusate volume = 500 ml*.

** *Time after start of NMP, values in median and IQR (interquartile range)*.

*GM-CSF, granulocyte-macrophage colony-stimulating factor; IFN, interferon; IL, interleukin; TNF, tumor necrosis factor*.

**Table 4B:** Luminex results^*^ stratified for urine recirculation and urine replacement.

	**Kidneys with urine recirculation (*****n*** **=** **8)**		**Kidneys without urine recirculation (*****n*** **=** **4)**		*p*-value^***^1 h	*p*-value^***^6 h
	1 h^**^	6 h^**^	1 h^**^	6 h^**^		
GM-CSF	420.6, 9,993	53.5, 2,300	10,000, 0	12.6, 27.7	0.11	0.21
IFN-γ	10,000, 0	10,000, 9,982	10,000, 0	10,000, 0	>0.9	0.42
IL-10	2,160, 8196	1,890, 3,727	5,023, 9,988	1,214, 3,607	>0.9	>0.9
IL-12p40	10,000, 0	10,000, 7,481	10,000, 0	10,000, 7,499	>0.9	0.83
IL-12p70	10,000, 0	10,000, 0	10,000, 0	10,000, 7,493	>0.9	>0.9
IL-1RA	1,214, 2,229	1,933, 2,710	453.7, 419.7	192.6, 969	0.68	0.26
IL-1α	10,000, 0	10,000, 9,935	5,010, 9,986	10,000, 0	0.09	0.42
IL-1β	5,082, 9,986	113.7, 7,534	10,000, 7,499	1.2, 3	0.67	0.02
IL-2	10,000, 9,998	19.7, 7,518	10,000, 0	5,001, 9,999	0.42	0.53
IL-4	10,000, 0	10,000, 0	10,000, 0	10,000, 0	>0.9	>0.9
IL-6	4,493, 9,040	8,496, 4,829	19.6, 47.7	5,224, 14,384	0.11	0.68
IL-8	66.1, 10,943	16,501, 35,438	23.9, 55.5	17,625, 9,744	0.77	0.89
TNF-α	18.4, 560.8	459.7, 1,277	8.9, 9.4	354.7, 221.3	0.46	0.37

* *Number of cells in pg/ml; overall perfusate volume = 500 ml*.

** *Time after start of NMP, values in median and IQR (interquartile range)*.

*** *P-value result of comparison with and without urine recirculation*.

*GM-CSF, granulocyte-macrophage colony-stimulating factor; IFN, interferon; IL, interleukin; TNF, tumor necrosis factor*.

### Donor Factors in Context With Perfusate Measurements

Perfusates of DBD kidneys had a significant lower number of leukocytes (median, IQR) after 6 h of NMP compared to DCD kidneys; 99,862 (178,223) in DBD vs. 466,163 (239,703) in DCD, *p* = 0.02. There were less T cells (median, IQR), CD4+ and CD8+ T cells, in the perfusates of DBD kidneys after 6 h of NMP compared to DCD organs; 30,071 (22,961) in DBD vs. 102,356 (56,002) in DCD, *p* = 0.01.

Donor hypertension did not correlate with any of the perfusate markers measured.

Estimated glomerular filtration rate (eGFR) of the kidney donors and their corresponding serum creatinine were related with changes of IFN-γ and IL-6 over 6 h NMP without reaching significance. Perfusate NGAL after 6 h of NMP was insignificantly higher in kidneys from a donor with higher serum creatinine at the time of retrieval.

Duration of CIT did not correlate with any of the biomarkers (NGAL, KIM-1, and L-FABP), neither with number of leukocytes nor with cytokines in the perfusate. The correlations coefficients (Spearman's rho) and associated *p*-values are shown in Table 5; *p* < 0.001 considered significant according to Bonferroni correction.

**Table 5:** Correlation of hemodynamic and metabolic parameters with perfusate biomarker, cells, and cytokines.

**Characteristic**	**Measured in perfusate**	**Spearman's rho**	***p*-value**
Donation after brain death	Leukocytes hour 6	−0.710	0.01
	Total T cells hour 6	−0.759	0.004
Donor age	GM-CSF hour 6	−0.655	0.021
	IFN-γ hour 6	0.588	0.04
	IL-1α hour 6	0.588	0.04
	IL-1β hour 6	−0.709	0.01
	NGAL hour 6	−0.718	0.009
	ΔNGAL^*^	−0.869	<0.001^*^
CVA as cause of death	ΔIL-1β	−0.641	0.03
	ΔIL-2	−0.599	0.04
	Non-classical monocytes hour 6	−0.717	0.009
Donor eGFR	ΔIFN-γ	0.624	0.03
	ΔIL-6	−0.629	0.03
	L-FABP hour 6	−0.662	0.02
	Δneutrophils	−0.615	0.03
	Median arterial flow	0.832	0.001
	Arterial flow hour 6^*^	0.860	<0.001^*^
	pH hour 6	0.592	0.04
Donor serum creatinine	ΔIFN-γ	−0.661	0.02
	NGAL hour 6	0.629	0.03
	ΔNK cells	0.671	0.02
	Median arterial flow	−0.839	0.001
	Arterial flow hour 6	−0.776	0.003
URC	Perfusion time	0.720	0.008
	IL-1β hour 6	0.718	0.009
Arterial flow hour 6	CD8+ cells hour 6	0.615	0.03
	Δneutrophils	−0.751	0.005
	%FoxP3 hour 6	−0.629	0.03
	TNF-α hour 6	0.627	0.03
	Median arterial flow^*^	0.881	<0.001^*^
	Lactate hour 6	−0.590	0.04
	Median lactate	−0.720	0.008
	pH hour 6	0.669	0.02
Perfusate pH hour 6	Median pH^*^	0.947	<0.001^*^
	Median arterial flow	0.746	0.005
	NGAL hour 6	−0.627	0.03
Hourly urine output	IL-1RA hour 6	−0.82	0.001
	Δclassical monocytes	−0.594	0.04
	KIM-1 hour 6	−0.804	0.002
	ΔKIM-1	−0.727	0.007
NGAL hour 6	ΔNGAL	0.839	0.001
	GM-CSF hour 6	0.657	0.02
	ΔGM-CSF	0.629	0.03
	IL-1β hour 6	0.599	0.04
	median lactate	*0.664*	0.02
ΔNGAL	IL-1α hour 6	−0.624	0.03
KIM-1 hour 6	IL-1RA hour 6	0.627	0.03
	IL-1α hour 6	0.624	0.03
	ΔIL-1β hour 6	0.609	0.04
	Δintermediate monocytes	0.594	0.04
	Δnon-classical monocytes	0.720	0.008
	ΔNK cells	0.608	0.04
L-FABP hour 6	IL-2 hour 6	−0.599	0.04
	ΔL-FABP	0.711	0.009
	eosinophils hour 6	−0.592	0.04
	macrophages hour 6	0.641	0.03
ΔL-FABP	IL-2 hour 6	−0.626	0.03
	Δeosinophils	−0.650	0.02
	Δ non-classical monocytes	−0.594	0.04

*Δ, delta (difference start-6-h perfusion value); URC, urine recirculation; CVA, cerebrovascular accident; GM-CSF, granulocyte macrophage-colony stimulating factor; IL, interleukin; IFN, interferon; NK, natural killer; NGAL, neutrophil gelatinase-associated lipocalin; L-FABP, liver-type fatty acid-binding protein; KIM-1, kidney injury molecule 1; TNF, tumor necrosis factor*.

*We applied the Bonferroni method to correct for multiple testing in the correlation analyses. N = 64 correlations were tested for statistical significance, consequently, the adjusted significance level is 0.05 divided by 64*.

* *P-values < 0.00078 (<0.001) were considered statistically significant*.

### NMP Perfusion Hemodynamics

Renal arterial flow in ml/min after 6 h of NMP correlated insignificantly with the CD8+ cell count. Overall, perfusates of kidneys with higher arterial flow had lower TNF-α levels. Kidneys from donors with higher eGFR at time of retrieval developed a significantly better arterial flow until hour 6 of NMP.

**Figure 3:**
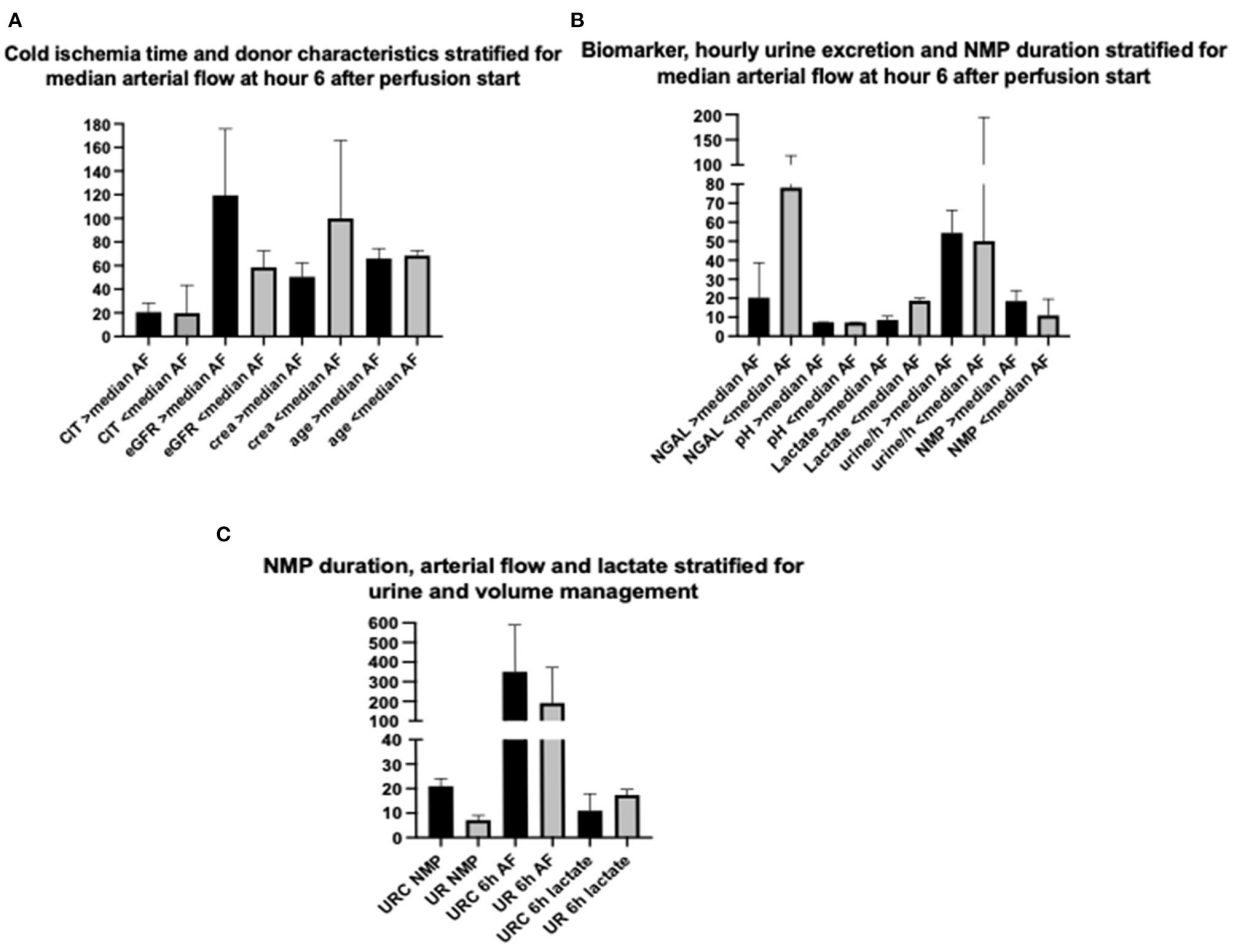
**(A)** Comparison of cold ischemia time (CIT), estimated donor glomerular filtration rate (eGFR), donor serum creatinine (crea) at time point of retrieval, and donor age (age) stratified for median arterial flow (303.5 ml/min) after 6 h of NMP start; depicted values (column) of parameters are in median and IQR. **(B)** Comparison of perfusate NGAL, perfusate pH, perfusate lactate at hour 6 after NMP start, hourly urine excretion, and NMP duration stratified for median arterial flow (303.5 ml/min) after 6 h of NMP start; depicted values (column) of parameters are in median and IQR. **(C)** Comparison of NMP duration, arterial flow at hour 6 after NMP start and perfusate lactate at hour 6 after NMP start stratified for perfusate volume management (urine recirculation URC of urine replacement UR); depicted values (column) of parameters are in median and IQR.

Figure 3A displays a comparison of CIT and donor characteristics stratified for median (IQR) arterial flow, 303.5 (186) ml/min at hour 6 after NMP start. There was no significant difference in duration of CIT (*p* = 0.7) and donor age (*p* = 0.8) for NMP kidneys reaching higher or lower arterial flow than the median of 303.5 ml/min. Donor eGFR (*p* = 0.002) was higher and donor serum creatinine lower (*p* = 0.002) in NMP kidneys reaching a higher arterial flow than the median.

Arterial flow after 6 h of NMP and median arterial flow correlated inversely. Perfusate lactate measured at hour 6 after NMP start was significantly lower in NMP kidneys reaching an arterial flow higher than the median of 303.5 ml/min; *p* = 0.004, shown in Figure 3B. The duration of NMP had no impact on reaching higher or lower median arterial flow; *p* = 0.6, depicted in Figure 3B.

Perfusate pH measured at hour 6 after NMP-start correlated significantly with median pH over time. There was a trend toward a more physiological pH and lower NGAL levels of kidney perfusates with higher arterial flow after 6 h of NMP. Figure 3B displays the relation of NGAL, pH and lactate, all measured at hour 6, with arterial flow. Kidneys with arterial flows higher than the median of 303.5 ml/min had non-significantly lower NGAL-perfusate levels (*p* = 0.07) and a more physiological pH (*p* = 0.06) but significantly lower perfusate lactate levels (*p* = 0.004).

NMP kidneys with higher arterial flow had non-significantly higher volume of hourly urine output; *p* = 0.7, shown in Figure 3B. There was also a link between KIM-1 levels after 6 h of NMP and the change of KIM-1 in the perfusate within the first 6 h of NMP. The correlations coefficients (Spearman's rho) and associated *p*-values are shown in Table 5; *p* < 0.001 considered significant according to Bonferroni correction.

### Volume Management

The application of URC led to significantly longer periods of NMP; *p* = 0.01, shown in Figure 3C. There were no other significant correlations of type of volume management, URC or UR, in regard to donor (type of donor, age, cause of death, serum creatinine, and eGFR), preservation (CIT, WIT), and hemodynamic factors (arterial flow during NMP). Perfusate lactate after 6 h of NMP was comparable between URC and UR kidneys; *p* = 0.6, displayed in Figure 3C.

The correlation coefficients (Spearman's rho) and associated *p*-values are shown in Table 5; *p* < 0.001 considered significant according to Bonferroni correction.

### Biomarkers of Renal Injury

After application of the Bonferroni correction for multiple testing, there were no significant correlation of NGAL, KIM-1, and L-FABP with other parameters measured in the perfusate. There was a connection between NGAL levels after 6 h of NMP and the concentration of GM-CSF and its change over time, ΔGM-CSF, in the perfusate. The concentration of IL-1β was non significantly higher in perfusates with higher 6-h NGAL levels. Perfusates with higher KIM-1 levels after 6 h of NMP had a higher count of non-classical monocytes. Perfusates with higher L-FABP levels at hour 6 after NMP start had also non-significantly more macrophages in the perfusate. The correlations coefficients (Spearman's rho) and associated *p*-values are shown in Table 5; *p* < 0.001 considered significant according to Bonferroni correction.

## Discussion

We herein report for the first time the results of measuring several biomarkers, including cytokines and leukocytes, in a normothermic human kidney perfusion model comparing a novel approach of urine recirculation (URC) to facilitate perfusate homeostasis and volume control (12, 13) with the technique of replacement of excreted urine using Ringer's lactate. Urine recirculation led to NMP durations up to 24 h and biomarker could be detected and analyses throughout these perfusions. The focus of this manuscript, however, was on the time point “hour 6” post perfusion start, as this was the latest comparable time point both, URC and UR NMP kidneys reached (17). Moreover, we focused on correlations with perfusion parameters and donor characteristics which are readily available at the time of decision making if a kidney is deemed transplantable after normothermic preservation and evaluation or declined for clinical use. This approach was chosen to examine some possible surrogate parameters, captured during perfusion, for organ viability. Our analyses were performed in a model of discarded human kidneys, therefore the important link to outcomes after successful transplantation is not available and no statements can be made in regard to estimating probable occurrence of delayed or primary non-function in the clinical setting.

The Cambridge group of Clatworthy, Ferdinand et al. (11), reported on NMP kidneys with higher inflammatory gene expression detectable in recipients who experienced prolonged DGF after receiving an NMP kidney. In their analyses, the course of 2 h NMP led to an upregulation of oxidative phosphorylation, but also an upregulation of a number of genes important for immune and inflammatory processes with NFkB induced TNF-α signaling as the major part of it (11). We detected an increase of TNF-α in perfusates in URC and UR kidneys, but the change over time was insignificant and more importantly, there was no difference at any time of perfusate TNF-α between the URC and UR kidneys. However, interestingly renal arterial flow (in URC and UR kidneys) was higher in kidneys with lower perfusate TNF-α which implicates a link between inflammatory potential of the perfusate and one of the best-studied hemodynamic parameters, especially in a pressure-fixed system in which increasing arterial flow over time is a parameter of kidney function (6, 18). In concordance with Ferdinand et al. (11), we also saw an increase of IL-8 in our perfusates over time. In line with IL-8, IL-6 increased over time in URC and UR kidneys without significant differences, but was not associated directly with any parameters identifying organ function during kidney NMP. However, both interleukins are known to be inflammatory with possible negative effects on renal parenchyma (19–21) and could be a future target for *ex-situ* organ treatment to prevent detrimental effects for the organ recipient. Our data revealed that IL-1β was higher in perfusates with higher content of NGAL and KIM-1 which are well-known markers for impaired kidney function and, NGAL at least, available to be measured in the clinical routine (2, 22–24). Another cytokine which could become of interest in future in a dynamic, normothermic preservation setting is GM-CSF. It is an immunregulatory cytokine which has been studied extensively recently due to its potential association with hyperinflammation in COVID-19 (25). GM-CSF is pro-inflammatory and plays a role in activation of macrophages and antigen-presenting cells (26). Whereas, in patients, as described in the literature, increased GM-CSF levels are associated with increased cytokines IL-6, TNF-α, IFN-γ, and IL-18 (25, 27), in our perfusates GM-CSF decreased over time. In perfusates with higher NGAL, GM-CSF was also higher compared to perfusates with lower NGAL which could potentially indicate less organ damage.

The Cambridge group did not detect any differences between DBD and DCD organs in terms of inflammatory gene signature they described. The only difference we could observe between DBD and DCD organs was the significant higher number of leukocytes, CD4+ and CD8+ T-cells in DCD-perfusates after 6 h of NMP compared to DBD organs; independent of URC or UR. This finding could be representing the cessation of blood flow in DCD organs and warm ischemia time prior to the start of retrieval, as the donor leukocytes have still a “route out” in DBD kidneys. In future, it will be interesting to compare DBD kidneys not only with DCD ones, but also with kidneys procured after normothermic regional perfusion.

The most relevant findings of our analyses were the clear connection of perfusate lactate, perfusate pH and urine output with several kidney function parameters in the donor as well as with published kidney injury markers as NGAL, KIM-1, and L-FABP (2). Perfusate lactate was lower the higher the arterial flow was and also, potentially important for defining timing for *ex-situ* organ assessment, the 6-h values of lactate, renal arterial flow and pH correlated with their respective median values. NGAL, a biomarker we would define as a routinely available biomarker (22), was also higher in perfusates with higher lactates and could be a surrogate biomarker for the donor kidney function on the circuit as it correlated significantly with donor creatinine and eGFR. Urine output during NMP, a marker implemented in the Hosgood and Nicholson score already, was associated positively with KIM-1 levels in the perfusate. In addition to our finding that biomarkers can be measured and correlated with transplant factors, overall NMP time itself did not correlate with any of the parameters measured. In particular, there was no association of a preservation period of 6 h and beyond with arterial flow and inflammatory potential of the perfusate. Such a finding could be crucial for implementing longer-term kidney NMP in the clinical routine.

Our results, gained from an *ex-situ* NMP setting cannot be correlated with clinical study results yet, but different cytokine and immune cell patterns do offer an important target to invest more research, particularly in forthcoming clinical use of the NMP device. The downside of performing cell and cytokine analyses solely in the perfusate and not in the tissue or from any other components in the circuit, is a limitation of our investigation, is the missing answer to the question of where the immune cells were possibly migrating to.

To summarize, clinically available perfusion parameters as perfusate lactate, pH and NGAL correlate well with donor characteristics, renal arterial flow, cytokines, immune cell changes, and KIM-1 in a discarded human NMP model. Potentially, lactate, pH and NGAL become a trinity to support decisions and fulfill the criteria to be diagnostic, predictive, and therapeutic biomarkers (28) in future for longer-term kidney NMP. In a non-transplant model long-term perfusion by applying URC was feasible and safe and also 6 h of NMP with UR seem to be applicable in a clinical transplant setting. Kidney NMP beyond 1 or 2 h might be helpful and instrumental in screening and discover markers indicating primary non-function of suboptimal organs (29). Therefore, these parameters should be considered as additional viability markers expanding the current decision-making score developed by Hosgood et al. (6).

## Data Availability Statement

The raw data supporting the conclusions of this article will be made available by the authors, without undue reservation.

## Author Contributions

AW designed the study, involved in the development of the perfusion device, performed the perfusions, collected and analysed the data, and wrote the manuscript. JS and JF performed the Luminex and FACS analyses. ML, JH, and RP were involved in data analyses, interpretation, and revision of the manuscript. CC and PF were instrumental for the study set-up, the development of the perfusion device, and revision of the manuscript. All authors contributed to the article and approved the submitted version.

## Funding

This research was supported by the Oxford Centre for Drug Delivery Devices under Programme Grant EP/L024012/1 from the UK's Engineering and Physical Sciences Research Council.

## Conflict of Interest

PF and CC are co-founders of OrganOx R Limited, receive consultancy payments as non-executive medical and technical directors of OrganOx R Limited, and are shareholders. JF is the Chief Scientific Officer of the ex-vivo Research Center CIC, but receives no payments and has no shares and is an executive director of Perfusion Biotechnology Limited. The remaining authors declare that the research was conducted in the absence of any commercial or financial relationships that could be construed as a potential conflict of interest.

## Publisher's Note

All claims expressed in this article are solely those of the authors and do not necessarily represent those of their affiliated organizations, or those of the publisher, the editors and the reviewers. Any product that may be evaluated in this article, or claim that may be made by its manufacturer, is not guaranteed or endorsed by the publisher.

## Supplementary Material

Supplementary Table 1A Flow cytometry results for urine recirculation (URC) for 30 min, 1, 6, 12, and 24 h after start of normothermic perfusion; 8 URC kidneys reached 12 h, 5 URC kidneys 18 h, and 4 URC kidneys 24 NMP h.

Supplementary Table 1B Luminex results for urine recirculation (URC) for 1, 6, 12, and 24 h after start of normothermic perfusion; 8 URC kidneys reached 12 h, 5 URC kidneys 18 h, and 4 URC kidneys 24 NMP h.

## Abbreviations

CIT: cold ischemia time
DBD: donation after brain death
DCD: donation after circulatory death
Δ: delta
ECD: extended criteria donor
ECMO: extracorporeal membrane oxygenation
ESRD: end stage renal disease
HMP: hypothermic machine perfusion
IRR: intrarenal resistance
KIM-1: kidney injury molecule-1
LDH: lactate dehydrogenase
L-FABP: liver-type fatty acid-binding protein
NADH: nicotinamide adenine dinucleotide
NGAL: neutrophil gelatinase-associated lipocalin
NMP: normothermic machine perfusion
SCS: static cold storage
WIT: warm ischemia time.
