# Supplementary material for: Hemodynamics and Metabolic Parameters in Normothermic Kidney Preservation Are Linked With Donor Factors, Perfusate Cells, and Cytokines
Source: Front Med (Lausanne). 2022 Jan 10;8:801098. doi: 10.3389/fmed.2021.801098 (PMC8784871; doi:10.3389/fmed.2021.801098)
Supplement: Supplementary Table 1A — Flow cytometry results for urine recirculation (URC) for 30 min, 1, 6, 12, and 24 h after start of normothermic perfusion; 8 URC kidneys reached 12 h, 5 URC kidneys 18 h, and 4 URC kidneys 24 NMP h. [file Table_1.docx]

| **Table S1A: Flow cytometry results*** | **Kidneys with urine recirculation (n=8)** | | | | | ***p-value*** 12 hours*** | ***p-value*** 24 hours*** |
| --- | --- | --- | --- | --- | --- | --- | --- |
|  | **30 minutes**** | **1 hour**** | **6 hours**** | **12 hours** (n=8/8)** | **24 hours** (n=4/8)** |  |  |
| Total leukocytes | 194687, 169462 | 301435, 173,632 | 285198, 314757 | 234431, 172513 | 170382, 154393 | *0.81* | *0.40* |
| Total T Cells | 39778, 42889 | 54203, 18377 | 79875, 79263 | 52959, 68535 | 49355, 53051 | *0.70* | *0.99* |
| CD4 T cells | 14579, 24059 | 24088, 14444 | 30468, 54969 | 28046, 29692 | 19366, 27624 | *0.88* | *0.99* |
| CD8 T cells | 16913, 13757 | 33008, 19555 | 31977, 21867 | 22723, 25189 | 26215, 19494 | *0.99* | *0.99* |
| NK T cells | 2943, 5681 | 4595, 3800 | 8050, 11446 | 8172, 11519 | 12531, 10029 | *0.99* | *0.99* |
| B cells | 17692, 29619 | 18707, 26760 | 31089, 43484 | 27360, 22744 | 19495, 33933 | *0.99* | *0.99* |
| Monocytes |  |  |  |  |  |  |  |
| Classical | 6310, 10731 | 18017, 19859 | 7188, 11711 | 3414, 2958 | 2672, 786 | *0.99* | *0.99* |
| Intermediate | 3462, 5310 | 9255, 11880 | 1363, 2799 | 978, 987 | 670, 1005 | *0.99* | *0.99* |
| Non-classical | 2743, 3285 | 7667, 6711 | 3790, 3645 | 2330, 2158 | 2393, 3412 | *>0.99* | *>0.99* |
| Eosinophils | 545, 12346 | 613.4, 5528 | 1122, 24421 | 655, 20381 | 961, 1158 | *0.99* | *0.99* |
| Neutrophils | 157, 202 | 379.1, 156.2 | 210.2, 351.4 | 477, 1130 | 80, 201 | *>0.99* | *>0.99* |
| NK cells | 11780, 56253 | 34918, 63636 | 26187, 48707 | 10157, 21305 | 6477, 59192 | *0.89* | *0.98* |
| Macrophages | 3073, 9088 | 4433, 11636 | 3709, 4719 | 8367, 12575 | 10250, 10743 | *0.99* | *0.99* |
|  |  |  |  |  |  |  |  |
| * number of cells in cells/ml; overall perfusate volume = 500ml | | | | | |  |  |
| ** time after start of NMP, values in median and IQR (interquartile range) | | | | | |  |  |
| *** comparison with 30 minutes value | | | | | |  |  |
|  |  |  |  |  |  |  |  |
|  |  |  |  |  |  |  |  |
