# Supplementary material for: Hemodynamics and Metabolic Parameters in Normothermic Kidney Preservation Are Linked With Donor Factors, Perfusate Cells, and Cytokines
Source: Front Med (Lausanne). 2022 Jan 10;8:801098. doi: 10.3389/fmed.2021.801098 (PMC8784871; doi:10.3389/fmed.2021.801098)
Supplement: Supplementary Table 1B — Luminex results for urine recirculation (URC) for 1, 6, 12, and 24 h after start of normothermic perfusion; 8 URC kidneys reached 12 h, 5 URC kidneys 18 h, and 4 URC kidneys 24 NMP h. [file Table_2.docx]

| **Table S1B: Luminex results*** | **Kidneys with urine recirculation (n=8)** | | | | ***p-value*** 12 hours*** | ***p-value*** 24 hours*** |
| --- | --- | --- | --- | --- | --- | --- |
|  | **1 hour**** | **6 hours**** | **12 hours** (n=8/8)** | **24 hours** (n=4/8)** |  |  |
| GM-CSF | 420.6, 9993 | 53.5, 2300 | 1053, 9959 | 2958, 7776 | *>0.99* | *>0.99* |
| IFN-γ | 10000, 0 | 10000, 9982 | 10000, 9958 | 10000, 7462 | *0.98* | *0.99* |
| IL-10 | 2160, 8196 | 1890, 3727 | 3460, 6565 | 8, 2816 | *0.99* | *0.97* |
| IL-12p40 | 10000, 0 | 10000, 7481 | 10000, 9976 | 10000, 0 | *0.91* | *>0.99* |
| IL-12p70 | 10000, 0 | 10000, 0 | 10000, 2498 | 10000, 7500 | *0.99* | *0.99* |
| IL-1RA | 1214, 2229 | 1933, 2710 | 1094, 1746 | 911, 2324 | *>0.99* | *>0.99* |
| IL-1a | 10000, 0 | 10000, 9935 | 5034, 9983 | 5161, 9688 | *0.87* | *0.92* |
| IL-1b | 5082, 9986 | 113.7, 7534 | 16, 2566 | 969, 7936 | *0.91* | *0.99* |
| IL-2 | 10000, 9998 | 19.7, 7518 | 8, 2572 | 5045, 9969 | *0.77* | *0.99* |
| IL-4 | 10000, 0 | 10000, 0 | 10000, 0 | 10000, 0 | *>0.99* | *>0.99* |
| IL-6 | 4493, 9040 | 8496, 4829 | 7098, 6210 | 4904, 6837 | *0.98* | *>0.99* |
| IL-8 | 66.1, 10943 | 16501, 35438 | 16185, 80275 | 8174, 16391 | *<0.0001* | *0.77* |
| TNF-α | 18.4, 560.8 | 459.7, 1277 | 504, 1997 | 1378, 4196 | *0.99* | *0.99* |
|  |  |  |  |  | *0.68* | *0.46* |
| * number of cells in pg/ml; overall perfusate volume = 500ml | |  |  |  |  |  |
| ** time after start of NMP, values in median and IQR (interquartile range) | | | | |  |  |
| *** comparison with 1 hour value | | | | | |  |
| GM-CSF=granulocyte-macrophage colony-stimulating factor; IFN=interferon; IL=interleukin; TNF=tumour necrosis factor | | | | |  |  |
